# A Federated Learning Latency Minimization Method for UAV Swarms Aided by Communication Compression and Energy Allocation

**DOI:** 10.3390/s23135787

**Published:** 2023-06-21

**Authors:** Liang Zeng, Wenxin Wang, Wei Zuo

**Affiliations:** 1 School of Cyberspace Science and Technology, Beijing Institute of Technology, No. 5 Zhongguancun South Street, Beijing 100081, China; 2 School of Computer Science and Technology, Beijing Institute of Technology, Beijing 100081, China; 1120190897@bit.edu.cn; 3 School of Automation, Beijing Institute of Technology, Beijing 100081, China; 1120201520@bit.edu.cn

**Keywords:** UAV network management, federated learning, latency, sustainability, energy consumption, communication resource optimization

## Abstract

Unmanned aerial vehicle swarms (UAVSs) can carry out numerous tasks such as detection and mapping when outfitted with machine learning (ML) models. However, due to the flying height and mobility of UAVs, it is very difficult to ensure a continuous and stable connection between ground base stations and UAVs, as a result of which distributed machine learning approaches, such as federated learning (FL), perform better than centralized machine learning approaches in some circumstances when utilized by UAVs. However, in practice, functions that UAVs must perform often, such as emergency obstacle avoidance, require a high sensitivity to latency. This work attempts to provide a comprehensive analysis of energy consumption and latency sensitivity of FL in UAVs and present a set of solutions based on an efficient asynchronous federated learning mechanism for edge network computing (EAFLM) combined with ant colony optimization (ACO) for the cases where UAVs execute such latency-sensitive jobs. Specifically, UAVs participating in each round of communication are screened, and only the UAVs that meet the conditions will participate in the regular round of communication so as to compress the communication times. At the same time, the transmit power and CPU frequency of the UAV are adjusted to obtain the shortest time of an individual iteration round. This method is verified using the MNIST dataset and numerical results are provided to support the usefulness of our proposed method. It greatly reduces the communication times between UAVs with a relatively low influence on accuracy and optimizes the allocation of UAVs’ communication resources.

## 1. Introduction

The application of aerial platforms such as unmanned aerial vehicle swarms (UAVs), also known as swarms of drones, is expanding quickly. UAVs are unmanned aircraft consisting of several single small, low-cost UAVs. By working in concert, UAVs have demonstrated a powerful capability to achieve significant advantages in missions that would be difficult for a single UAV to accomplish. With their unique advantages, including high mobility and flexibility, UAVs have played an important role in many areas [1], including rescue, signal detection, terrain mapping [2,3,4,5,6], etc. The expanding prospects of the applications of UAVs has attracted a significant amount of attention from academia and industry. However, due to the flying height and mobility of UAVs, it is very difficult to ensure a continuous and stable connection between ground base stations and UAVs. Therefore, UAVs are better suited to perform tasks using distributed machine learning approaches than centralized machine learning approaches.

A distributed machine learning method called federated learning (FL) deconstructs data silos and unleashes the potential of AI applications [7]. FL enables the participants to achieve joint modeling by exchanging only encrypted intermediate results of machine learning without disclosing the underlying data and their encrypted form. Such a distributed FL approach can be well suited for UAV communication: after each UAV in the cluster has individually trained a model based on the data it has collected, it uses an intra-cluster network to share FL parameters with other UAVs. This not only reduces volume of communication between UAVs but also avoids the disclosure of sensitive data and protects privacy security to a certain extent.

A number of recent works have investigated the feasibility of FL-based UAVs communication, these aerial access networks are also regarded to be very important in the upcoming sixth-generation (6G) wireless systems [8,9,10]: Ref. [11] built a leader–follower mode UAVs-FL architecture for the first time, and discussed how wireless factors such as bandwidth and UAV angle deviation affect FL convergence. They also minimized the convergence cycle of UAVs-FL. The limited resources that a single UAV can carry also limit the performance of UAVs: when UAVs perform complex tasks, the pressure of the self-organized data interaction network increases with the expansion of the scale of the task model, and the communication energy consumption increases. In reference [12], path gain was examined depending on the distance between the UAVs using a ray tracing method in various scenarios and with various antenna types in an air-to-air communication channel during communication for two UAVs, one of which was considered to be a receiver and the other as a transmitter, with direct vision between them. Ref. [13] improved the task allocation mechanism of UAVs, effectively reduces communication energy consumption, and ensures model performance. Under various constraints of power control, transmission time, accuracy, bandwidth allocation, and computing resources, Ref. [14] minimized the overall energy consumption of each UAV with limited bandwidth. Ref. [15] maximized the transmission rate and improved the probability of successful data transmission based on deep Q-network (DQN), a convolutional block attention module (CBAM), and the value decomposition network (VDN) algorithm. Ref. [16] built a synchronous federated learning (SFL) structure for multi-UAVs and also performed a comparative analysis of asynchronous federated learning (AFL) and SFL. Ref. [17] reformulated the optimization problem as the framework of a Markov decision process (MDP) and designed a DRL-based algorithm to solve the MDP. Refs. [18,19] proposed a secure transmission approach with energy efficiency in UAV networks to deal with the crucial challenges of energy saving and security in UAV wireless networks. Refs. [20,21] suggested some new frameworks for distributed learning for sharing the model parameters that use less energy while maintaining good test accuracy performance.

However, there still exists a problem with these edge-oriented distributed machine learning approaches, which is that the communication demand is often very high, resulting in high communication consumption [22]. A typical parallel SGD model was designed and implemented through research, with a parameter matrix size of 2,400,000, running on a distributed parameter server system with one server node and 10 work nodes, and an Ethernet bandwidth of 1 Gbps between nodes. Under the above model and hardware configuration conditions, 60,000 sample data from the MNIST handwritten digit recognition dataset were used as input, and each sample underwent only one iterative training. The final complete training took 23 h, and it was found that 80% of the time and energy were spent on parameter exchange between the parameter server and the working node [23]. Thus, reducing communication consumption is a valuable entry point for improving such distributed machine learning methods.

In reality, the tasks that UAVs need to deal with are often very sensitive to latency, such as dynamic target recognition, emergency obstacle avoidance, etc. [24]. This latency sensitivity will directly affect the completion of the task and should be one of the primary considerations for optimizing the network management of UAVs. Based on this pilot–follower mode of UAVS-FL architecture, Ref. [25] proposed a non-orthogonal multiple access (NOMA) based UAVs-FL framework to jointly optimize the uplink and downlink transmission duration of the model and UAV power, aiming for minimization of the latency of a FL iteration round until a specified accuracy is reached. In addition, while considering the convergence, reliability, and latency-sensitivity requirements of UAVs, the constraints on the energy consumed by learning, communication and flight during FL convergence should also be considered. However, at present, most of the research on task allocation of unmanned aircraft clusters focuses on non-real-time tasks, and there is still a lack of more complete solutions for task allocation that consider both latency and reliability [26]. Therefore, motivated by the above reasons, this paper proposes a relatively complete solution for the situation where UAVs perform such latency-sensitive tasks.

It is worth mentioning that in the field of federated learning for edge computing, there is a similar problem: the network and node computing load are too heavy [27]. In large-scale training scenarios, a large amount of communication bandwidth is often required for gradient switching, which will greatly increase the cost of network infrastructure. At the same time, the limited computing resources of the edge nodes will also lead to higher overall latency, lower throughput, and intermittent poor connections in the model. Ref. [28] proposed a method for distributed machine learning to save communication resources named lazily aggregated gradient (LAG). This is a communication-efficient variant of stochastic gradient descent (SGD), which can adaptively skip gradient calculation based on the current gradient, and help reduce the communication and computing burden. Later, in order to further improve the performance in the random gradient scenario, lazily aggregated stochastic gradients (LASG) was proposed, which further reduces the communication bandwidth requirements, and the convergence rate is equivalent to the original SGD [29]. In addition to the idea of skipping redundant communication rounds, there have also been studies devoted to skipping some nodes in a certain round to ultimately achieve the purpose of saving communication resources. Ref. [30] proposed an efficient asynchronous federated learning mechanism (EAFLM) for edge network computing which compresses the redundant communication between the node and the parameter server in the training process according to the adaptive threshold to further reduce the communication consumption.

Based on the UAVs-FL model proposed in Ref. [11], we construct a leader–follower framework. On this basis, we have established an optimization problem and proposed a resource scheduling planning method specifically for the class of tasks that UAVs perform in practice, the latency-sensitive tasks. Our primary goal is to minimize communication latency in a federated learning round. Overall, the primary contributions of this paper are:

1. Introduction of the efficient asynchronous federated learning mechanism (EAFLM), which compresses communication times by up to 92.5% compared to the original communication times and minimizes the risk of private data leakage.
2. Establishment of an optimization problem with the aim of minimizing FL latency. Although this problem is non-convex, we have transformed it into two convex subproblems related to the transmit power and the CPU frequency of UAVs. By introducing the ant colony optimization (ACO) algorithm to plan the power allocation of UAVs, lower global latency can be achieved for latency-sensitive tasks. The FL iteration latency per round can also be reduced to 48.9% of the similar method.
3. In the MNIST dataset, the accuracy of machine learning tasks remained above 95%, which did not decrease compared to the situation without introducing the scheduling strategy in this paper.

In summary, in order to achieve a shorter global latency, the strategy initially allocates a portion of time and energy for local operations and subsequently plans for the power allocation methods, and ultimately achieves a reduced global latency.

The remainder of this paper is organized as follows: Section 2 describes the system model and gives out the problem model. Section 3 elaborates on the EAFLM-ACO strategy and the implementation of our proposed algorithm. In Section 4, simulations and analyses are presented to prove the efficiency of our proposed method. Section 5 summarizes this article.

## 2. System Model

**Figure 1:**
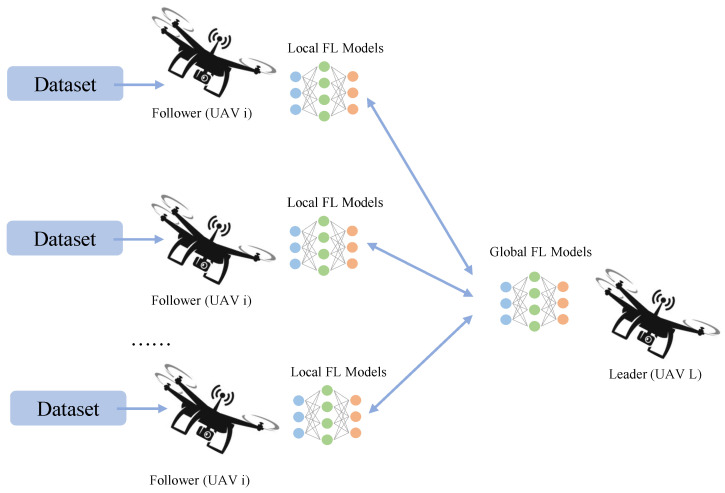
An illustration of the UAVs-FL architecture.

To study UAV network management based on FL, this chapter establishes a model as follows: a single group of UAVs consists of a leader UAV and *I* follower UAVs, with the follower UAVs forming the set I. The leader UAV is denoted as UAV L, and each follower UAV is denoted as UAV i (i∈I). The UAV group maintains a specific formation in the air, flying at a constant speed in the same direction at a certain altitude. The leader UAV and follower UAVs utilize FL to cooperate, performing machine learning tasks such as trajectory planning and target recognition. The overall architecture is shown in Figure 1.

### 2.1. Federated Learning Model

Use w to represent the global model parameters of UAV L, and $w_{i}$ represents the local model parameters of UAV i(i∈I). The size of the model parameters is defined as $S(w_{i})$. $N_{i}$ is the amount of sample data of UAV i. Assuming that each UAV i has a input sample set $\left\{x_{i1},x_{i2},\cdots ,x_{iN_{i}}\right\}$, and every $x_{in}$ only corresponds to one output $y_{in}$ through model $w_{i}$, which means the output set is $\left\{y_{i1},y_{i2},\cdots ,y_{iN_{i}}\right\}$ [31]. Take $D_{i}$ as the local sample set of UAV i, which means $D_{i}=\left\{\left(x_{i1},y_{i1}\right),\left(x_{i2},y_{i2}\right),\cdots ,\left(x_{iN_{i}},y_{iN_{i}}\right)\right\}$. The loss function $f(w_{i},x_{in},y_{in})$ reflects the predicted loss results of each sample. For every UAV i, the local loss function $F_{i}\left(w\right)$ on its sample set $D_{i}$ can thus be represented as the average of the loss function of each sample, and the global loss function F(w) is the weighted average of all local loss functions, that is:

(1) $$F\left(w\right)=\sum_{i\in I}\frac{N_{i}F_{i}w}{N}=\frac{1}{N}\sum_{i\in I}\sum_{n\in D_{i}}f\left(w_{i},x_{in},y_{in}\right),$$

The purpose of federated learning is to find a parameter model that minimizes the global loss function above. To achieve this optimal model, traditional centralized machine learning algorithms require all follower UAVs to upload their datasets to the leader UAV for centralized training. In the federated learning circumstances described in this paper, the following five steps are performed in a certain round [32].

1. Local gradient calculation: each UAV i computes its local gradient $g_{i}\left(t\right)$ at moment t based on its own local dataset $D_{i}$ and quantizes the gradient as follows:
  (2) $$g_{i}\left(t\right)={▽}_{w}F_{i}\left(w\left(t\right)\right),$$
2. Local gradient upload: after quantizing the local gradient, each UAV i establishes a communication link with UAV L to upload its local gradient.
3. Global gradient aggregation: UAV L weights and averages the gradients uploaded by each UAV m and obtains the aggregated gradient g(t) as follows:
  (3) $$g\left(t\right)=\frac{1}{N}\sum_{i\in I}N_{i}g_{i}\left(t\right),$$
4. Global gradient update: UAV L updates the parameters of the aggregated gradient using the method of gradient descent, where w(t+1) represents the global model parameters of iteration round *t* + 1, η represents the learning rate and η>0:
  (4) w(t+1)=w(t)−ηg(t),
5. Global parameter broadcast: UAV L broadcasts the updated global model parameters to all other UAV i. Each UAV i obtains the latest parameters and updates its local parameters for the next round of iterative learning.

In a federated learning system, these five steps are repeated until the maximum number of rounds is reached.

### 2.2. Communication Model

We assume that every follower UAV i in this FL iteration forms a group and communicates with UAV L using its local training model $w_{i}$. In Section 3.1, the selection procedure for determining which follower UAV participates in this iteration will be explained in detail. We assume that UAV L utilizes the index in the group as the decoding order for uploading the local model parameters to UAV L. We use $p_{i}$ to represent the transmit power of UAV i, i.e., the transmit power for uploading its data to the leader UAV. According to Shannon’s formula, we can represent the uplink data rate $R_{i}^{\mathrm{up}}$ between UAV i and UAV L as:

(5) $$R_{i}^{\mathrm{up}}=B_{i}^{\mathrm{up}}\log_{2}\left(1+\frac{p_{i}g_{i}}{\sum_{j=1}^{i-1}p_{j}g_{j}+B_{i}^{\mathrm{up}}\gamma_{0}}\right),\forall i\in I,$$

where $B_{i}^{\mathrm{up}}$ represents the uplink bandwidth, $p_{i}\in \left(0,p_{max}\right)$ represents the signal power of UAV i, $g_{i}$ is the channel power gain from UAV i to UAV L, and $\gamma_{0}$ is the spectral power density of the background noise.

After receiving model parameters uploaded by the follower UAVs, UAV L performs local model aggregation. Once the aggregation is complete, the updated global model is broadcast to all follower UAVs. Considering the follower UAV with the weakest channel power obtained from the leader UAV, the downlink data rate $R^{\mathrm{down}}$ from the leader UAV to the follower UAV with the weakest channel power gain can be expressed as:

(6) $$R^{\mathrm{down}}=B^{\mathrm{down}}\log_{2}\left(1+\frac{p_{L}\min_{i\in I}\left\{h_{i}\right\}}{B^{\mathrm{down}}\gamma_{0}}\right),$$

where $B^{\mathrm{down}}$ represents the downlink bandwidth, $p_{L}\in \left(0,p_{max}\right)$ represents the signal power of UAV L, $h_{i}$ is the downlink channel power gain from the UAV L to UAV i, and $\gamma_{0}$ is the spectral power density of the background noise.

Once the uplink and downlink data transmission rates of the channel are determined, the transmission latency can be calculated by the ratio of the size of the model parameters $S(w_{i})$ to the data transmission rate $R_{i}^{\mathrm{up}}$ or $R^{\mathrm{down}}$.

### 2.3. Latency Analysis

As previously stated, our goal is to reduce end-to-end latency by optimizing the latency. In this section, we calculate the main types of latency in a single communication round [32].

#### 2.3.1. Local Time Consumption of Follower UAVs

The total time consumption of follower UAVs can be divided into two parts, local gradient computation $T_{i}^{computation}$ and local gradient upload $T_{i}^{upload}$. They can be expressed as:

(7) $$T_{i}^{computation}=\frac{\sum_{n=1}^{N_{i}}S\left(x_{in}\right)c}{f_{i}}$$

(8) $$T_{i}^{upload}=\frac{S(w_{i})}{R_{i}^{\mathrm{up}}}$$

where $\sum_{n=1}^{N_{i}}S\left(x_{in}\right)$ represents the size of collected data for UAV i, *c* represents the workload of CPU cycles per data bit, $f_{i}\in \left(f_{min},f_{max}\right)$ represents the CPU frequency of UAV i, $S(w_{i})$ represents the total data size of UAV i corresponding to the local parameter gradient, and $R_{i}^{\mathrm{up}}$ represents the uplink data rate.

#### 2.3.2. Global Time Consumption of Leader UAV

The total time consumption of a leader UAV can also be divided into two parts: global gradient computation $T_{L}^{aggregation}$ and broadcast $T_{L}^{broadcast}$. They can be expressed as:

(9) $$T_{L}^{aggregation}=\frac{\sum_{i=1}^{I}S\left(w_{i}\right)\alpha }{f_{L}}$$

(10) $$T_{L}^{broadcast}=\frac{S(w_{i})}{R^{\mathrm{Down}}}$$

where α represents the computational complexity, $f_{L}\in \left(f_{min},f_{max}\right)$ represents the CPU frequency of UAV L, *I* represents the total number of devices involved in model aggregation, and $R^{\mathrm{down}}$ represents the downlink data rate.

#### 2.3.3. Total Time

For UAV L, it must first wait for the local gradient to be uploaded by the follower UAVs before starting gradient aggregation and model broadcast. This implies that the total latency of a round of federated learning is the sum of the longest local time consumption among all follower UAVs and the global time consumption of the leader UAV. Therefore, for a swarm of UAVs, the total latency of a complete federated learning round is:

(11) $$T=\max_{i\in I}\left\{T_{i}^{computation}+T_{i}^{upload}\right\}+T_{L}^{aggregation}+T_{L}^{broadcast}$$

### 2.4. Energy Consumption Model

In this paper, we only consider the computation energy consumption, communication energy consumption, and maneuvering energy consumption related to federated learning and communication between UAVs. The energy consumption of the follower UAVs and the leader UAV can be expressed by the following formulae, respectively:

(12) $$\begin{array}{cc} E_{i} & =E_{i}^{computation}+E_{i}^{upload}+E_{i}^{maneuvering}\\ \; & =\kappa f_{i}^{\mu }T_{i}^{computation}+p_{i}T_{i}^{upload}+\delta \left(T_{i}^{computation}+T_{i}^{upload}\right) \end{array}$$

(13) $$\begin{array}{cc} E_{L} & =E_{L}^{aggregation}+E_{L}^{broadcast}+E_{L}^{maneuvering}\\ \; & =\kappa f_{L}^{\mu }T_{L}^{aggregation}+p_{L}T_{L}^{broadcast}+\delta \left(T_{L}^{aggregation}+T_{L}^{broadcast}\right) \end{array}$$

where κ and μ represent the energy consumption efficiency and are both positive constants [25] and δ represents the average maneuvering power.

### 2.5. Optimization for Minimizing Latency

We take the transmit power and CPU frequency of each UAV as optimization variables and optimize the time of each round of federated learning to minimize it. So, we can establish the following optimization problem, referred to as Problem 1.

(14) $$\begin{array}{cc} \left(P1\right) & \min_{p_{i},f_{i},p_{L},f_{L}}T\left(t\right),\\ \;s.t.\; & c1:0⩽p_{i}⩽p_{\max},i\in I,\\ \; & c2:f_{\min}⩽f_{i}⩽f_{\max},i\in I,\\ \; & c3:0⩽p_{L}⩽p_{\max},\\ \; & c4:f_{\min}⩽f_{L}⩽f_{\max},\\ \; & c5:E_{i}⩽E_{i}^{max},i\in I,\\ \; & c6:E_{L}⩽E_{L}^{max}. \end{array}$$

where T(t) is the total time per round, which is the goal of the optimization problem. By controlling the transmit power of follower UAV $p_{i}$, CPU frequency of follower UAV $f_{i}$, transmit power of leader UAV $p_{L}$, and CPU frequency of leader UAV $f_{L}$, the single-round latency is minimized. The constraints include the transmit power and CPU frequency ranges of the UAVs. The energy consumption of follower UAV $E_{i}$ and leader UAV $E_{L}$ should also be lower than the maximum energy limit $E^{max}$.

## 3. The Proposed Method

In the previous section, we have established an optimization problem that minimizes the federated learning time per round by considering the transmit power and CPU frequency of the UAV as variables. In this section, we propose a resource optimization configuration scheme that combines EAFLM and ACO. The goal is to achieve the minimum communication latency in each round.

### 3.1. UAVs Network Management Based on EAFLM

A complete federated learning framework includes a parameter server and several learning nodes corresponding to UAV L and UAV i in this model. In each round *t*, the learning nodes obtain the global model w(t−1), compute the local gradient ${▽}_{m}\left(w\left(t-1\right)\right)$, and upload it to the server. The server aggregates the gradients, executes the optimization algorithm to update the model parameters, and then broadcasts the updated model parameters to each learning node. To minimize the need for establishing communication links, in this paper, we locally select learning nodes and allow some of them to skip certain rounds of communication. Here, we introduce the concept of ‘lazy nodes’ [30]. A lazy node is defined as a node that contributes less to the global gradient in a particular round of global gradient aggregation. In other words, the participation or exclusion of these nodes in a specific round of global gradient aggregation has almost no impact on the final result. Therefore, ignoring these nodes in this round of aggregation can have a good effect on the model performance. The set of lazy nodes satisfies:

(15) $$\frac{\left|\right|{▽}_{I_{neg}}^{t-1}{\left|\right|}^{2}}{I_{neg}}\leq \frac{\left|\right|{▽}_{I}^{t-1}{\left|\right|}^{2}}{I}$$

where ${▽}_{I}^{t-1}$ represents the total gradient uploaded by all followers within round t−1, ${▽}_{I_{neg}}^{t-1}$ represents the total gradient uploaded by all lazy nodes within round t−1, $I_{neg}$ is the size of the lazy node set, and *I* is the total number of follower UAVs.

In this paper, we optimize the global model using the gradient descent algorithm.

(16) $$w\left(t\right)=w\left(t-1\right)-\eta \nabla_{I}^{t-1}$$

where w(t) represents the global model parameters of iteration round *t*. η represents the learning rate.

Therefore,

(17) $${∥{▽}_{I_{neg}}^{t-1}∥}^{2}\leq \frac{I_{neg}}{\eta^{2}I}{∥w(t)-w(t-1)∥}^{2}$$

Because the global model tends to converge, the following approximation is used:

(18) w(t)−w(t−1)≈w(t−1)−w(t−2)

According to the mean inequality, we have:

(19) $$\begin{array}{cc} {∥\nabla_{I_{neg}}^{t-1}∥}^{2} & ={∥\sum_{i\in I_{neg}}\frac{S\left(x_{in}\right)\nabla_{i}^{t-1}}{\sum_{n=1}^{N_{i}}S\left(x_{in}\right)}∥}^{2}\\ \; & ⩽\frac{I_{neg}}{{\sum_{n=1}^{N_{i}}S\left(x_{in}\right)}^{2}}\sum_{i\in I_{neg}}{∥S\left(x_{in}\right)\nabla_{i}^{t-1}∥}^{2}, \end{array}$$

where $\sum_{n=1}^{N_{i}}S\left(x_{in}\right)$ represents the size of collected data for UAV i.

Let $I_{neg}=\left(1-\beta \right)I$. This implies that (1−β) represents the proportion of lazy nodes, which do not participate in communication, among all follower UAVs. Therefore, β represents the participation rate, which is the proportion of follower UAVs that participate in communication. If Equation (20) is satisfied, Equation (15) is also satisfied.

(20) $${∥S\left(x_{in}\right)\nabla_{i}^{t-1}∥}^{2}⩽\frac{{\sum_{n=1}^{N_{i}}S\left(x_{in}\right)}^{2}}{\eta^{2}I^{2}\left(1-\beta \right)}{∥w\left(t-1\right)-w\left(t-2\right)∥}^{2}$$

In summary, in each round *t*, UAV i locally verifies whether it satisfies Equation (20). If it does, the current round of upload will be skipped.

In the extreme case where all nodes in a particular round *t* satisfy Equation (20), UAV L will not receive the model information uploaded by any follower UAV. In such cases, UAV L selects a follower UAV randomly to participate in the upload after a specified time interval ΔT. This ensures that the federated learning task can continue in a relatively efficient situation. The specific time interval ΔT can be determined based on different scenarios. In this paper, we set ΔT as:

(21) $$\Delta T\geq \bar{T_{i}^{computation}+T_{i}^{upload}},i\in I$$

As for the time consumption in these extreme rounds, assuming that the device selected to the device is $i_{0}\left(i_{0}\in I\right)$, the latency for this round can be defined as:

(22) $$T=T_{i_{0}}^{computation}+T_{i_{0}}^{upload}+T_{L}^{aggregation}+2*T_{L}^{broadcast}+\Delta T$$

### 3.2. Latency Minimization Based on ACO

In the previous section, we formulated an optimization problem to minimize the time consumed in each round of federated learning by optimizing the transmit power and CPU frequency of each UAV. Next, we will solve this optimization problem based on ant colony optimization (ACO) Algorithm 1.

To solve the optimization problem, we will decompose it into sub-problems that will be solved independently through mathematical derivation and simplification. In Problem 1, the total time is defined as local time consumption and global time consumption. We have also calculated the time consumed for each step in the previous section. Due to the separate control of follower UAVs and the leader UAV, we will divide Problem 1 into two sub-problems: one focuses on the latency consumption of the follower UAVs, and the other focuses on the latency consumption of the leader UAV. Therefore, this optimization problem can be rewritten as Problem 2 and Problem 3 as follows.
**Algorithm 1:** ACO for minimized the latency in one round.**Input**:
   UAVs communication parameters: *I*, $N_{i}$, $S(x_{in})$, $S(w_{i})$, *c*, $g_{i}$, $\gamma_{0}$, $c_{L}^{\prime }$, $h_{i}$,$B_{i}^{\mathrm{up}}$ or $B^{\mathrm{down}}$;**Output**:   Best solution of $p_{i}$, $f_{i}$ or $p_{L}$, $f_{L}$ and the minimum latency;Initialize the model parameters: the size of ant colony *N*, pheromone value τ, pheromone evaporation coefficient ρ, pheromone weight α, transfer factor weight β, total pheromone release *Q*;Randomly initialize N ant solutions and pheromone value τ;Take iteration times as *k*;**while** *k < max iteration times* **do**  Obtain the best index and its τ;  **for** *each individual in the colony* **do**   Calculate the transition probability by
τ(t+n)=(1−ρ)·τ(t)+Δτ;  **end**  **for** *each individual in the colony* **do**  Update individual locations using local search and global search;  Determine whether an individual can move based on the restriction   condition and penalty function [33];  The penalty function is calculated as:  $PF\left(x\right)=k\sqrt{k}\sum_{i}\theta \left(p_{i}\left(x\right)\right)*p_{i}{\left(x\right)}^{\gamma^{p(x)}},$
  where $p_{i}\left(x\right)=max\left(0,g_{i}\left(x\right)\right),$
  θ is the multi-stage assignment function,
  γ
 depends on specific cases.
  Calculate the pheromone value;  Record the minimum latency with the solution;  **end****end**

Problem 2 represents the follower’s latency consumption:

(23) $$\begin{array}{cc} \left(P2\right) & \max_{i}\min_{p_{i},f_{i}}\frac{\sum_{n=1}^{N_{i}}S\left(x_{in}\right)c}{f_{i}}+\frac{S(w_{i})}{B_{i}^{\mathrm{up}}\log_{2}\left(1+\frac{p_{i}g_{i}}{\sum_{j=1}^{i-1}p_{j}g_{j}+B_{i}^{\mathrm{up}}\gamma_{0}}\right)},\\ \;s.t.\; & c1:0⩽p_{i}⩽p_{\max},\\ \; & c2:f_{\min}⩽f_{i}⩽f_{\max},\\ \; & c3:\left(\kappa f_{i}^{\mu }+\delta \right)\left(\frac{\sum_{n=1}^{N_{i}}S\left(x_{in}\right)c}{f_{i}}\right)+\left(p_{L}+\delta \right)\frac{S(w_{i})}{B_{i}^{\mathrm{up}}\log_{2}\left(1+\frac{p_{i}g_{i}}{\sum_{j=1}^{i-1}p_{j}g_{j}+B_{i}^{\mathrm{up}}\gamma_{0}}\right)}⩽E_{i}^{max}. \end{array}$$

where κ and μ represent the energy consumption efficiency and are both positive constants. δ represents the average maneuvering power. $\sum_{n=1}^{N_{i}}S\left(x_{in}\right)$ represents the size of collected data for UAV i. *c* represents the workload of CPU cycles per data bit. $B_{i}^{\mathrm{up}}$ represents the uplink bandwidth. $g_{i}$ is the channel power gain from UAV i to UAV L. $\gamma_{0}$ is the spectral power density of the background noise.

Problem 3 represents the leader’s latency consumption:

(24) $$\begin{array}{cc} \left(P3\right) & \min_{p_{L},f_{L}}\frac{\sum_{i=1}^{I}S\left(w_{i}\right)\alpha }{f_{L}}+\frac{S(w_{i})}{B^{\mathrm{down}}\log_{2}\left(1+\frac{p_{L}\min_{i\in I}\left\{h_{i}\right\}}{B^{\mathrm{down}}\gamma_{0}}\right)},\\ \;s.t.\; & c1:0⩽p_{L}⩽p_{\max},\\ \; & c2:f_{\min}⩽f_{L}⩽f_{\max},\\ \; & c3:\left(\kappa f_{L}^{\mu }+\delta \right)\left(\frac{c_{0}\sum_{m=1}^{M_{0}}\left|g_{m}\right|+c_{L}^{\prime }}{f_{L}}\right)+\left(p_{L}+\delta \right)\frac{S(w_{i})}{B^{\mathrm{down}}\log_{2}\left(1+\frac{p_{L}\min_{i\in I}\left\{h_{i}\right\}}{B^{\mathrm{down}}\gamma_{0}}\right)}⩽E_{L}^{max}. \end{array}$$

where $B^{\mathrm{down}}$ represents the downlink bandwidth, $p_{L}\in \left(0,p_{max}\right)$ represents the signal power of UAV L, and $h_{i}$ is the downlink channel power gain from the UAV L to UAV i.

Therefore, this simplified optimization problem can be solved by the Algorithm 1 above.

### 3.3. Overall Architecture

**Figure 2:**
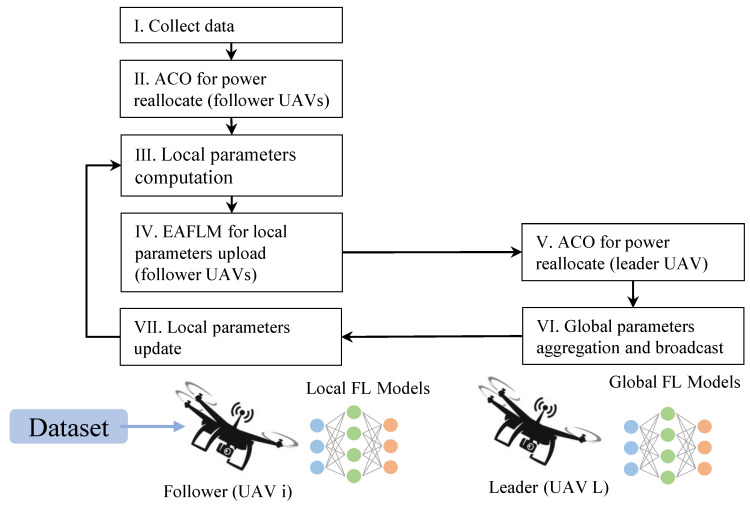
Overall architecture of the system.

The overall architecture is shown in Figure 2.

## 4. Results and Discussion

**Figure 3:**
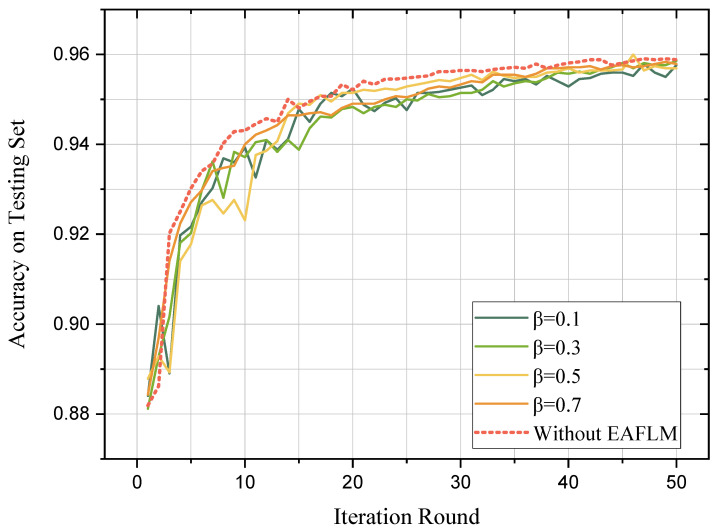
Convergence of accuracy.

**Figure 4:**
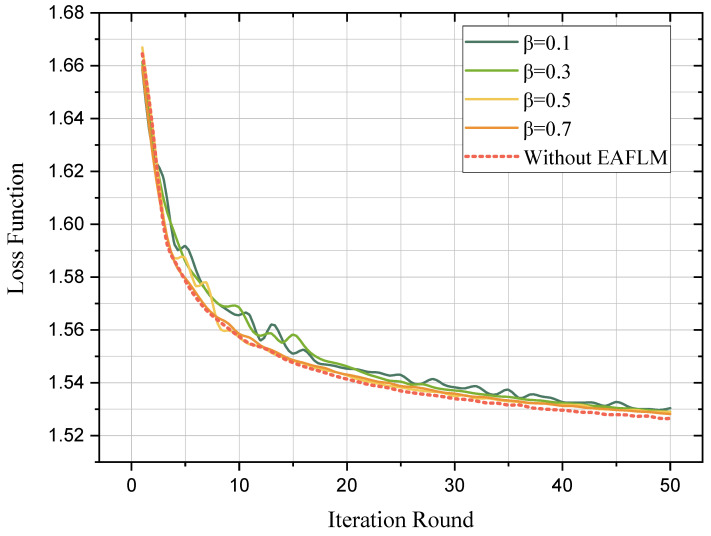
Convergence of loss function.

In this section, we verify the validity of our EAFLM scheme through numerical results. Specifically, we utilized the TensorFlow framework to construct a leader–follower UAVs-FL model comprising a leader UAV and nine follower UAVs. The follower UAVs are distributed in a circle centered around the leader UAV. The UAVs maintain the same constant speed while moving and a fixed distance from each other, which means that their power consumption for maneuvering can be roughly considered as the same constant. Meanwhile, the channel power gain $g_{i}$ between the leader UAV and the follower UAV during FL can also be roughly considered as a constant. We test the performance of the proposed method on the handwritten numeric dataset MNIST. Among them, 10% of the data are retained as the test set of the global model, and a three-layer MLP (multi-layer perceptron) neural network is used as the model of the classification task for the machine learning task of recognizing handwritten digits. The simulation parameters are as Table 1 [25]:

Firstly, the accuracy and loss function of classification results were evaluated, shown in Figure 3 and Figure 4, respectively. The experimental results show that the proposed method EAFLM-ACO can achieve FL convergence in 50 rounds.

It can be observed that as the value of β decreases (indicating fewer follower UAVs participating in each round of federated learning theoretically), the accuracy curve and loss function curve exhibit more fluctuations before reaching convergence. The introduction of the EAFLM strategy introduces some instability to the federated learning model because the participation of follower UAVs in communication is not fixed for each round. However, after 50 rounds, all five models with different β values reached convergence, and their accuracies were similar to each other. Therefore, it can be concluded that the proposed method in this paper reduces the scale of communication while ensuring the training results.

However, it should be noted that the research focus of this paper is the communication in the federated learning framework, and the model structure and optimization algorithm have not been studied too much. Therefore, the reasons behind the overfitting problems and other problems in the experiment in this paper and their solutions do not belong to the scope of this paper. Similarly, experimental indexes such as accuracy are only for the purpose of comparing the performance of various methods, rather than evaluating the merits and demerits of the model. Moreover, because the comparative experiment of different methods adopts the same configuration, it can be said that the indexes in the experiment have the value of comparison.

**Figure 5:**
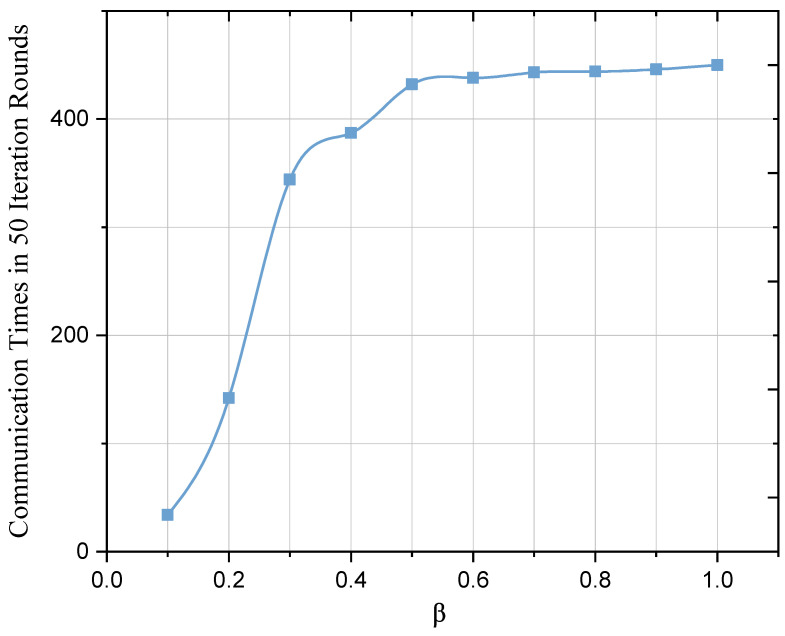
Communication times between the different values of β.

Because of the EAFLM strategy, after performing gradient calculation, UAV i makes an additional local check to see if it meets the conditions of skipping the round. If so, this UAV i skips this round of communication. It can be clearly seen from the results in Figure 5 that when β is within the range of 0.1 to 0.3, the communication times have a very obvious change. When β is greater than 0.3, the slope of the curve decreases gradually. In other words, when β is below 0.3, the communication times of the UAVs-FL model will be significantly compressed compared to the case without communication compression (β=1).

**Figure 6:**
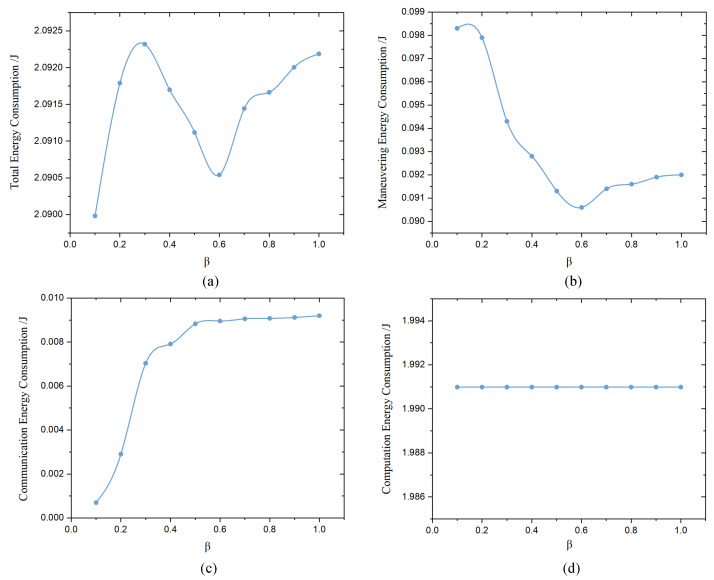
Average energy consumption of follower UAVs between the different values of β. (**a**) Total energy consumption. (**b**) Maneuvering energy consumption. (**c**) Communication energy consumption. (**d**) Computation energy consumption.

Figure 6 illustrates the average energy consumption of each follower UAV over 50 iterations of completing a federated learning task. Specifically, Figure 6a shows the total energy consumption, whereas Figure 6b–d represent the maneuvering energy consumption, communication energy consumption, and computation energy consumption, respectively. Because our optimization objective primarily aims to minimize the latency of federated learning, which directly affects the flight duration of the UAV fleet, the maneuvering energy consumption fluctuates due to different values of β. With the introduction of our EAFLM strategy, which compresses communication times among UAVs, the communication energy consumption becomes proportionate to the average communication times, as the energy consumption per unit time for communication is constrained to a similar level. Consequently, the average communication energy consumption decreases due to the significant compression of communication times. Regarding computation energy consumption, each UAV is required to perform the local gradient computation and update steps in each iteration, resulting in a consistent level of computation energy regardless of changes in β. However, it should be noted that this energy consumption variation can not infer the conclusion that this method is energy efficient.

We also compared our proposed method with a similar existing study in Table 2. The NOMA (non-orthogonal multiple access) is an FL framework designed for UAVs. The optimization goal of this method is also to achieve the minimum delay for each FL round, while using uplink transmission durations, downlink broadcasting duration, and CPU frequency as controllable variables however. Under the same environmental parameters, our method achieved a 48.9% improvement in reducing latency compared to NOMA. This indicates that our optimization problem, which uses UAVs’ CPU frequency and communication power as optimization variables, holds promise for further investigation.

**Figure 7:**
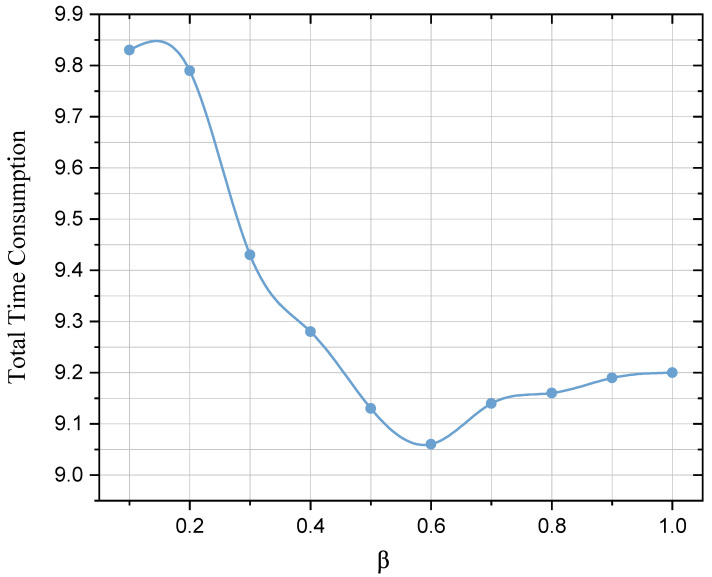
Total time consumption of 50 iteration rounds with different values of β.

Finally, we analyze the total time latency required for the FL model to reach convergence, as shown in Figure 7. We can see that in the case without communication compression (β=1), the total time consumption is much lower than the case when times of communication are greatly compressed (β<0.3). This is because the highly compressed communication times are likely to lead to a situation in which there is no follower UAV in a certain round that meets the conditions to participate in the communication. The UAVs will waste δT waiting for the leader UAV to check if it is an extreme case, where the leader UAV will then randomly select a follower UAV to receive its upload parameters, which thus undoubtedly becomes a waste of time. It is also worth mentioning that the total communication time is significantly reduced when β is around 0.6. This implies that an appropriate degree of communication compression holds substantial significance for latency control.

In summary, this method effectively lowers the latency of an individual round of FL by compressing communication times and reallocating transmit power and CPU frequency.

## 5. Conclusions

In this paper, a leader–follower architecture UAVs-FL model is constructed. On this basis, an optimization problem is established for latency-sensitive tasks in UAVs. The EAFLM-ACO method is proposed with the main goal of achieving the shortest communication latency possible. Our method significantly compresses communication times among UAVs, ensures low latency in FL iterations, and optimizes the allocation of UAV communication resources. The model accuracy is also taken into account.

EAFLM-ACO significantly reduces communication times between UAVs while maintaining a relatively low impact on accuracy. After the follower UAVs train the local model, they check whether they meet the conditions for participating in this round of communication according to the self-inspection conditions. If they meet the conditions, the gradient will be uploaded to the leader UAV. This selective gradient exchange approach also mitigates the risk of disclosing private data. At the same time, the allocation of the transmit power and CPU frequency is adjusted locally to achieve the shortest latency.

The effectiveness of this method is additionally verified by experiments. As the degree of communication compression increases, the number of rounds required from FL model to achieve convergence are nearly the same and the accuracy and loss function of machine learning tasks are not significantly different from those without compression. In order to minimize the latency, the transmit power and CPU frequency are reallocated. The latency of each FL iteration is reduced by 48.9% compared to other similar methods.

As for the further work, considering that the UAVs perform computationally heavier tasks or the amount of local data increases further, then at the end of each round of calculation, the additional gradient check will further increase the calculation time consumption, which may affect the energy allocation of the whole UAVs group. In order to reduce the pressure of local computation, a “check-free” mechanism can be invented, which may help to reduce the computation work caused by gradient checking. In future work, we will conduct further research on the “inspection exemption” strategy.

## Figures and Tables

**Table 1:** Simulation parameters.

Parameters	Value
Number of follower UAVs, $I_{0}$	9
Uplink bandwidth, $B^{\mathrm{up}}$	5MHz
Downlink bandwidth, $B^{\mathrm{down}}$	5MHz
Noise power density, $\gamma_{0}$	−174dBm/Hz
Uplink channel gain, $g_{i}$	$2\times 10^{-7}$
Downlink channel gain, *h*	$1\times 10^{-7}$
Workload of CPU cycles per data bit, *c*	70 CPU cycle/bit
Computational complexity, α	50
Size of local model, $S(w_{i})$	1.8 MB
Size of global model, S(w)	1.3 MB
Size of collected data, $\sum_{n=1}^{N_{i}}S\left(x_{in}\right)$	1.8 MB
UAV CPU frequency limit, $\left(f_{min},f_{max}\right)$	$\left[0.2\;\mathrm{GHz},0.4\;\mathrm{GHz}\right]$
UAV transmit power limit, $\left(p_{min},p_{max}\right)$	0.3 w
Average maneuvering power, δ	0.01 w
Leader UAV energy limit, $E_{L}^{\max}$	0.1 J per round
Follower UAV energy limit, $E_{i}^{\max}$	0.04 J per round
Learning rate, η	0.01 [32]
Energy consumption efficiency, κ, μ	$10^{-28}$, 2

**Table 2:** Performance comparison.

Method	Minimum Latency for a FL Round
EAFLM-ACO	0.27693 ^1^
EAFLM-PSO	0.36232
NOMA	0.54267 [25]
Baseline scheme	0.57168 ^2^

^1^ When $p_{i}$ takes 0.02072w, $f_{i}$ takes 0.27462GHz, $p_{L}$ takes 0.196902w and $f_{L}$ takes 0.399971GHz, the optimal solution is obtained. ^2^ In baseline scheme, $p_{i}$ takes 0.01w, $f_{i}$ takes 0.3GHz, $p_{L}$ takes 0.15w and $f_{L}$ takes 0.35GHz.

## Data Availability

Not applicable.

## Abbreviations

The following abbreviations are used in this manuscript:

UAVs	Unmanned Aerial Vehicle swarms
FL	Federated Learning
EAFLM	Efficient Asynchronous Federated Learning Mechanism
ACO	Ant Colony Optimization
